# Electrochemical Modeling Applied to Intercalation Phenomena Using Lattice Kinetic Monte Carlo Simulations: Galvanostatic Simulations

**DOI:** 10.3390/e27070663

**Published:** 2025-06-20

**Authors:** E. Maximiliano Gavilán-Arriazu, Andrés Ruderman, Carlos Bederian, Eduardo Moran Vieyra, Ezequiel P. M. Leiva

**Affiliations:** 1 Instituto de Bionanotecnología del NOA (INBIONATEC), Universidad Nacional de Santiago del Estero (UNSE), Santiago del Estero G4206XCP, Argentina; edu_fac2001@yahoo.com.ar; 2 Laboratorio de Energías Sustentables, Facultad de Matemática, Astronomía, Física y Computación, Universidad Nacional de Córdoba, Córdoba X5000GYA, Argentina; andres.ruderman@unc.edu.ar; 3 Instituto de Física Enrique Gaviola, Consejo Nacional de Investigaciones Científicas y Técnicas (CONICET), Córdoba X5000GYA, Argentina; carlos.bederian@unc.edu.ar; 4 Instituto de Fisicoquímica de Córdoba (INFIQC), Consejo Nacional de Investigaciones Científicas y Técnicas (CONICET), Córdoba X5000GYA, Argentina

**Keywords:** kinetic Monte Carlo, intercalation, lithium-ion batteries, sodium-ion batteries, alkali-ion batteries, lattice-gas model

## Abstract

In the present work, we address the theory of the lattice-gas model to the study of intercalation materials by using a novel kinetic Monte Carlo (kMC) algorithm for the simulation of an electrochemical method of everyday use in R&D laboratories: constant-current chrono-potentiometric measurements. The main aim of the present approach is to show how to use these atomistic simulations to study intercalation materials used as electrodes in alkali-ion batteries under galvanostatic conditions. The framework can be applied to related areas. To accomplish this, we explain the electrochemical background, linking the continuum scale with the microscopic events of discrete simulations. A comprehensive theoretical approach developed in a previous work is used as a reference for this aim. The galvanostatic kMC algorithm proposed is explained in detail and is subject to validation tests. The present work may serve as a basis for future implementations of kMC under galvanostatic conditions to study phenomena beyond the applicability of simulations on the continuum scale.

## 1. Introduction

Lithium-ion batteries (LIBs) are undoubtedly the energy storage systems that have changed the energy paradigm worldwide. However, the global demand for lithium and the development of technologies such as electric vehicles require batteries with higher storage capacity and shorter charging times. The industry is also looking for alternatives to traditional LIBs, such as sodium-ion batteries [1,2,3], which are already a reality, and other alkaline metal batteries. This is why the careful optimization and the innovative development of electrode materials are essential.

**Figure 1:**
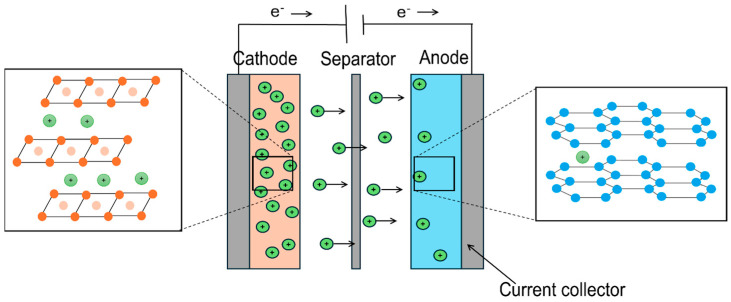
Typical configuration of a lithium-ion battery cell.

Any battery based on metal-ion technology basically has the same type of operation: a cathode and an anode where the ions are intercalated during the charge and discharge cycles of the electrochemical cell; a separator that prevents electrical contact between the electrodes; and an electrolyte, which is the phase (usually liquid) through which the electrons migrate from one electrode to the other (Figure 1).

Most of the key physical phenomena that determine the charge level and the charging rate of the battery occur in the active materials of the electrodes. This is why, in the last years, nanoelectrochemical measurements have become of special relevance. This type of measurement allows for the study of “bare” materials at a single-particle level, that is, in the absence of conductive additives or binders that are incorporated in the assembly of battery electrodes. This allows for a detailed physicochemical study of the factors that determine the properties of materials to be used as electrodes, and a subsequent more efficient design of them. A comprehensive discussion on single-particle measurements and the modeling devoted to understanding these systems has been given very recently in reference [4]. This modeling has been usually based on a continuum approach, that is, the solution of partial differential equations describing the diffusion of lithium ions within the materials, coupled to a charge-transfer equation describing the insertion/disinsertion of these ions into/out of the active material [5,6,7]. Though useful, this model has several approximations among which an important one is the absence of phase transitions. Thus, comparative calculations with more sophisticated models are necessary to check the impact of this approximation on the predicted behavior. One of these models involves the use of a lattice approach to describe the position of the inserted ions, calculating the evolution of the system according to the Kinetic Monte Carlo (kMC) scheme.

kMC simulations have proven to be a valuable tool for studying the materials of intercalation and surface-level batteries [8,9,10]. Numerous applications have been reported, such as the study of the growth and reaction mechanism of the SEI [11], of the dynamics of solid and liquid electrolytes [12,13], and of post-Li-ion batteries [14,15]. The importance of this methodology lies in the fact that it allows for long-term simulations (on the time scale of the intercalation phenomena) with a microscopic detail of the processes. This is something that is not feasible with other simulation techniques (such as Molecular Dynamics) given the high computational costs. For example, in constant-current chrono-potentiometric (galvanostatic) experiments, the charging rate of the electrochemical cell is expressed as the C-rate (C). A value of 1C is the charging rate necessary to charge the cell in one hour, 2C for half an hour, and so on. Thus, the charging of materials, depending on the volume of them, can occur on the scale of seconds to hours.

In the literature, few works are performing kMC simulations under constant current conditions [16], which is surprising given that these measurements are routinely used in R&D laboratories for the study of materials. In the present work, we propose a new algorithm for this purpose, providing a tool for future improvements of the microscopic model, to account for the physics behind different materials. For example, apart from the long time scale simulations already mentioned, kinetic Monte Carlo offers advantages compared with other simulation methodologies, such as the treatment of the phase coexistence of intercalation compounds or the ability to deliver information about the entropic variations due to the concentration of ions inside the material.

kMC has become a popular technique through the years, especially for simulating diffusion processes for both diffusion on surfaces and in the bulk of the materials [17,18,19,20,21,22,23,24,25]. However, if one wants to emulate the physics of intercalation, it is essential to incorporate the kinetics of the insertion/exit of ions at the electrode/electrolyte interface. This is key since the performance of materials depends on the coupling between diffusive and interface phenomena, making this a complex problem to study separately. In previous studies, we have developed a model for this purpose, devoted to the study of lithium-ion intercalation in graphite under potentiostatic and voltammetric conditions [26,27,28]. It has been shown that, in this system, both thermodynamics, which dictates the interactions between inserted ions, and kinetics play a very important role. In that work, to bridge the gap between continuum description and microscopic modeling under potentiostatic signals (validation of kMC simulations), we compared kMC simulations with a theoretical work by Montella [29], showing excellent agreement. A similar approach is developed here to validate the kMC algorithm.

In physics, the use of scalable parameters to reduce a problem from many dimensions to only a few is very popular. With this idea in mind, and seeking to reveal the role of finite diffusion and interface kinetics in the loading capacity and rate of materials, we have recently developed a theoretical framework at the continuum level for the lithiation of materials under galvanostatic conditions, using a single-particle model [5,7,30]. In essence, the concept of this approach is similar to that of Aoki et al. [31] for voltammetry measurements and that of Montella [29] for potentiostatic measurements. In the present work, this model is used as a reference to test the physics of kMC simulations. We will go briefly into this theoretical framework in the “Scalable Parameters at Galvanostatic Conditions” section.

The present work is organized as follows: In the Section 2, we show in detail the physics of intercalation for continuum-scale models and for kinetic Monte Carlo, as well as the equations linking the macro- and microscopic parameters. The Section 3 shows the validity of the kMC model when subjecting the system to different conditions to demonstrate the importance of considering both the statistics and the limitations arising from the system’s kinetics in the design of simulations. Finally, the Section 4 summarizes the most important points of this work and provides an overview of future applications of the proposed methodology.

## 2. Theory and Methods

The redox reaction taking place in the intercalation phenomena can be represented with the following equation:

(1) $$M^{+}+\langle *\rangle +e^{-}\;\begin{array}{c} k_{f}\\ ⇌\\ k_{b} \end{array}\;\langle M^{+}\rangle$$

Here, $M^{+}$ is an ion of an alkaline metal; 〈∗〉 is an intercalation site of the electrode material, located at the electrode/electrolyte interface; $e^{-}$ is the elemental charge; $\langle M^{+}\rangle$ is the intercalated metal ion inside the material; and $k_{f}$ and $k_{b}$ are the rate constants for the forward and the backward redox process, respectively. This interfacial phenomenon is one of the key factors influencing the intercalation of ions. It depends on several factors, like the crystalline structure and exposed crystalline face of the material, the type of electrolyte, the spurious reactions that may form stable fouling films, the metal ion’s solvation/desolvation properties, and the metal ion’s size.

Once the ion is intercalated, it can move randomly inside the solid without causing substantial changes to its structure. This phenomenon, denoted as ion diffusion, strongly depends on the chemical nature of the material, its crystalline structure, and the kind of intercalated ion. Ion diffusion is characterized by the thermodynamic property denominated diffusion coefficient D and also depends on the number of ions that have been intercalated in the material (occupation) [32]. This shows a complex scenario where interfacial phenomena are coupled to diffusive ones and both are affected by the thermodynamics and kinetics of the problem. This requires adequate modeling of the physics of the processes taking place.

In this sense, kinetic Monte Carlo has proven to be a technique that incorporates the complex framework of the phenomena, allowing for the study of the intercalation process in different materials. Curiously, to date, few articles have addressed the intercalation phenomenon incorporating all its complexity. Furthermore, to the best of our knowledge, only one article has studied the response of the potential of the electrode when applying a constant current signal within the kMC approach, given that this type of experimental signal is commonly used in R&D laboratories for the study of intercalation materials and their application in power sources devices. This is why, in the present work, we focus on the kMC approach to intercalation phenomena in potentiometric experiments and its validation with continuum models.

A complete review of the application of kMC to lithium batteries and other systems can be found in reference [8].

### 2.1. Continuum Single-Particle Model

Since the results of the kMC simulations will be compared with those of a continuum approach, we will revisit the latter within the denominated single-particle model (SPM). There, the diffusion of ions inside active particles (solid phase) is considered using Fick’s laws for planar diffusion:

(2) $$\frac{\partial x}{\partial t}=D\left(\frac{\partial^{2}x}{{\partial r}^{2}}\right)$$

In this equation, $x=\frac{C_{M^{+}}}{C_{s,max}}\;$is the ion fractional concentration inside the particle located at (0≤x≤1), where $C_{M^{+}}$ is the concentration of ions and $C_{s,max}$ is the maximum concentration; D is the diffusion coefficient inside the host material; and r is the distance from the surface (r=d) to the center of the slab (r=0).

Von Neumann boundary conditions are considered at the frontier points of the space grid:

(3) $${\left(\frac{\partial x}{\partial r}\right)}_{r=d}=-\frac{i_{c}}{FDC_{s,max}}$$

(4) $${\left(\frac{\partial x}{\partial r}\right)}_{r=0}=0$$

Here, $i_{c}$ is the constant current density applied.

The Butler–Volmer approach, Equation (5), is used to describe charge transfer kinetics at the electrode/electrolyte interface:

(5) $$i_{c}=i_{0}\left\{exp\left[\frac{\left(1-\alpha \right)F\left(\phi -\phi^{0}(x_{s})\right)}{RT}\right]-exp\left[\frac{-\alpha F\left(\phi -\phi^{0}(x_{s})\right)}{RT}\right]\right\}$$

Here, the exchange current density is given by the following:

(6) $$i_{0}=Fk^{0}C_{s,max}{x_{s}}^{\alpha }{\left(1-x_{s}\right)}^{1-\alpha }$$

In these equations, ϕ is the electrode potential, $\phi^{0}$ is the equilibrium potential at the fractional occupation $x_{s}$, $k^{0}$ is the heterogeneous rate constant for charge transfer, and α is the transfer coefficient (here considered α=1/2). We use the subscript s to highlight the fact that the Butler–Volmer equation contains the fractional occupation at the surface of the intercalating material. The symbols F, R, and T have their usual meanings.

Under constant current conditions, the charging rate ($I_{c}$) satisfies the following relationship:

(7) $$I_{c}=\frac{Q}{t}$$

where t is time and Q is a volumetric capacity in Coulomb. The charging rate is generally expressed as a C-rate ($C_{r}$), instead of $I_{c}$, in the analysis of battery materials.

(8a) $$C_{r}=\left|\frac{I_{c}t_{h}}{Q_{max}}\right|$$

Here $C_{r}$ is the C-rate, an integer or fractional positive number; $Q_{max}$ is the maximum capacity of the particle (maximum amount of charge that a given material can store); and $t_{h}$ is the time for an hour. If $Q_{max}$ is expressed in Coulomb units, $t_{h}$ is considered as 3600 s.

As shown, in this model, the voltage profiles depend on four parameters: D, $k^{0}$, d, and $C_{r}$. It is also important to define the State-of-Charge (SoC). In the present framework, the SoC represents the average ion occupancy within the particle and is calculated as a normalized capacity according to the following:

(8b) $$SoC=\frac{Q}{Q_{max}}$$

A SoC ≈ 0 means an empty particle, while a SoC ≈ 1 corresponds to the maximum occupation of ions in the material.

#### Scalable Parameters at Galvanostatic Conditions

**Figure 2:**
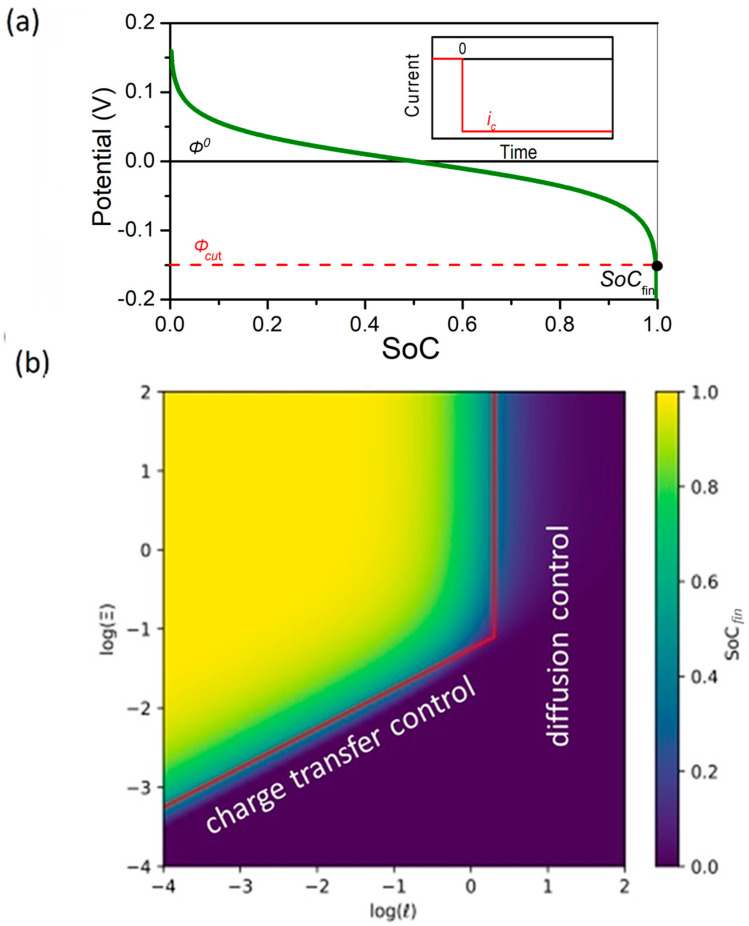
(**a**) Scheme of the potential response as a function of the State-of-Charge (SoC) under the application of a galvanostatic transient, according to the current perturbation given in the inset. The potential/SoC transient shown corresponds to the reversible limit, with fast diffusion of the inserted ions. The figure also illustrates the cut-off potential $\phi_{cut}$ used to determine the final State-of-Charge ${SoC}_{fin}$. (**b**) Galvanostatic color map of the final State-of-Charge reached by the electrode ${SoC}_{fin}$ (see Figure 2a for definition) as a function of the scaled parameters Ξ and l, taken from Gavilán-Arriazu et al. [4], with permission.

As described in the previous section, the performance of an SP with planar geometry in the model considered is determined by four parameters: D, $k^{0}$, d, and $C_{r}$. This means that the galvanostatic response of a material, illustrated in Figure 2a, depends on the combination of these four parameters. We briefly discuss the nature of this curve in the case of a lithiation taking place close to equilibrium conditions ($i_{c}$ close to 0 in Equation (5)). We represent a negative current transient ($i_{c}<0,\;$inset) applied to an empty particle, so that at t = 0 (x=Q=SoC=0), we start to fill the material with Li-ions. The transient starts with a low lattice occupation, at high potentials. As lithiation proceeds, the potential decreases, the lattice half being occupied at the potential $\phi^{0}\left(\frac{1}{2}\right)=0.$ Then, when lithiation further proceeds, the electrode potential goes towards minus infinity, as demanded by the Nernst equation in the limit where $x_{s}\to 1$.

The use of scalable parameters defined from D, $k^{0}$, d, and $C_{r}$ allows us to reduce the dimensionality of the problem. In the work of reference [5], we proposed two scalable parameters: a kinetic parameter Ξ (Equation (9)) and a finite-diffusion one l (Equation (10)), which reduces the problem to a two-dimensional one instead of a four-dimensional one.

(9) $$\Xi =k^{0}{\left(\frac{t_{h}}{C_{r}D}\right)}^{1/2}$$

(10) $$l=d^{2}\left(\frac{C_{r}}{Dt_{h}}\right)$$

Considering the definitions of Ξ and l, it is clear that Ξ is characterized by the rate constant $k^{0}$ and l by the diffusion length d. Thus, changing $k^{0}$ will switch the value of Ξ, which corresponds to a situation where the kinetics of the electrochemical reaction becomes faster or slower, depending on $k^{0}$. On the other hand, varying the particle size d will change l, and thus larger values of d will be linked to greater diffusional limitations. This is, briefly speaking, the physical meaning of Ξ and l. For a more detailed explanation please see reference [4].

However, this is a more complex problem since the model depends on the four parameters D, $k^{0}$, d, and $C_{r}$, as mentioned previously. Using Ξ and l reduces the dimensionality of the problem since the potential profiles under galvanostatic conditions (electrode potential vs. SoC representation) will depend only on Ξ and l, and on the cut-off potential chosen, $\phi_{cut}$, as it was shown in reference [4].

Regarding $\phi_{cut}$, we consider it as a constant value in the present work. We have shown in references [4,5] that for a Langmuir intercalation isotherm and an equilibrium potential $\phi^{0}=0\;V$, the particle reaches almost the full State-of-Charge, SoC = 0.997, at $\phi_{cut}=-0.15\;V$. We will use this $\phi_{cut}$ value in the present simulations.

Master curves or maps for the analysis of single-particle materials used as electrodes in LIBs can be constructed with this approach [5,7,30]. The basis of the methodology is simple: The Ξ and l space is explored for the same $\phi_{cut}$ and the final SoC reached in each point of this space is saved. The final SoC is called here ${SoC}_{fin}$ (see Figure 2a) and can be represented in a 3D or a color map, where ${SoC}_{fin}=f(\Xi$, l). A representation of ${SoC}_{fin}$ vs. log(Ξ) and log(l) is shown in Figure 2b.

The first thing that stands out is that there is a high-capacity (SoC) zone of greater than 80% (yellow zone) and a poor SoC zone of less than 10% (blue zone). In a recent work, we have shown that one important feature of this map is that it presents two borderlines that define capacity drop edges due to different phenomena. These borders are marked with red lines in Figure 2. Below log(*l*) = 0.3, a borderline is defined by a straight line with slope ½. This line was related to a SoC drop due to charge transfer limitations at the electrode/solution interface. On the other hand, the vertical line at log(*l*) = 0.3, up to log(Ξ) = −1, was related to a SoC drop due to diffusion limitations. This is analyzed in detail in reference work [4].

The use of this map simplifies the analysis of the single-particle materials, since we only need two dimensionless parameters to know their final State-of-Charge, at a given cut-off potential. Thus, this map serves as a guide to evaluate how different limitations affect kMC simulations.

### 2.2. Lattice Kinetic Monte Carlo Simulations

#### 2.2.1. Kinetic Monte Carlo Scheme

A kMC simulation performs a chain of Poisson events that are characterized by a hierarchy of rate constants (Γ). In this hierarchy, events with higher rates are more likely to occur than those with lower rates [33].

The rate constants calculated for all possible events for a given lattice configuration are stored in a list, from which one is chosen based on a random number $\xi_{1}$ ranging between 0 and 1. After selecting and allowing the event to proceed, time t is updated by an increment Δt. This time increment is determined by an exponential distribution, given in Equation (11), using a second random number $\xi_{2}$ in the same range of $\xi_{1}$.

(11) $$\Delta t=-\frac{ln(\xi_{2})}{\sum_{i}\Gamma_{i}}$$

The *rejection-free algorithm* used in this work ensures that an event takes place at each time step without any rejection. Once the time is updated, all possible events are calculated from the newly generated configuration, and the procedure is repeated. To finish the simulation, a cut-off criterion is required, which will be detailed later, when we discuss how to adapt the algorithm to potentiometric measurements.

As will be seen in more detail in the next sections, the ordinary events considered in an intercalation material are intercalation, deintercalation, and diffusion. Transport processes for the motion of ions through the electrolyte, outside the material, may also be introduced; this is not the case in the present work. Other events or restrictions can be easily added, and this is conditioned by the model that one wants to build to emulate a particular chemical physics scenario.

#### 2.2.2. Slab Model and kMC Events

**Figure 3:**
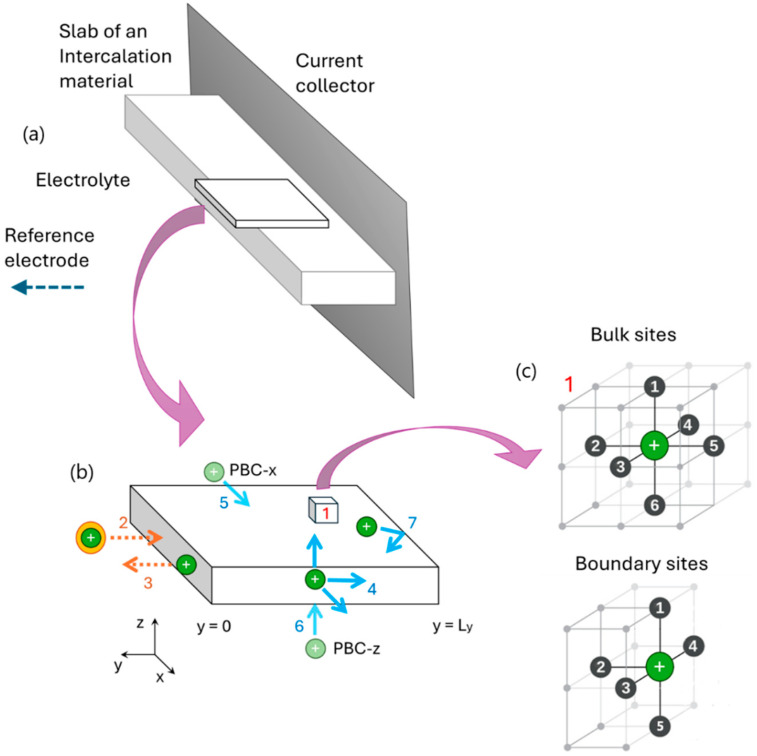
Schematic representation of the present model of the electrochemical cell at different scales: (**a**) macroscopic view, (**b**) atomic scale. In these representations, different features of the kMC model are labeled with a number, and the explanation of these features is detailed in the main text, while (**c**) details of the unit cell used, showing the number of neighboring sites for a site located in the bulk of the material and at the boundaries.

The model of the electrochemical hemi-cell within the SPM is shown in Figure 3. Figure 3a shows a general picture; the slab is located between a current collector and the electrolyte solution. The reference electrode is located at some point in the direction indicated with the dashed blue arrow. One of the faces of the slab is in contact with the electrolyte solution, colored with light gray. A section of the slab is marked with black lines. This section is represented in more detail in Figure 3b. The lattice kinetic Monte Carlo model employed for simulating this section of the slab is detailed there. A 3D lattice-gas was constructed with a simple cubic crystal structure where ions can be placed; this is marked with 1 in Figure 3b and detailed in Figure 3c. The number of intercalation sites at each axis are $N_{x}$, $N_{y}$, and $N_{z}$, and so the total number of sites (that defines the capacity of the section) is calculated as $N_{T}=N_{x}\times N_{y}\times N_{z}$. In the case of monovalent ions, the maximum capacity of the slab sectional material is then $Q_{max}=eN_{T}$, where e is the elemental charge in Coulombs. The bulk sites have 6 nearest neighbors, while boundary sites located at y = 0 and y = $L_{y}$ have 5 nearest neighbors, as shown in Figure 3c.

The simulations considered diffusion, as well as intercalation and deintercalation events. To mimic the real picture of an electrode material in an electrochemical cell, the following conditions were imposed:

- The electrode was set in contact with a reservoir of ions at the x–z plane located at y = 0, so the lithium ions can be intercalated (marked with 2 in Figure 3a) or deintercalated (marked with 3) at the boundary sites of this plane. The concentration of ions in the electrolyte was constant.
- Diffusion of ions was allowed to the first unoccupied neighboring lattice sites (marked with 4).
- Periodic boundary conditions (PBC) were imposed in the x-direction (marked with 4) and along the *z*-axis (marked with 6).
- The diffusional motion of ions was restricted to the length of the simulation box along the *y*-axis (marked with 7).
- A metal lithium reference electrode was considered, so that the potential of the working electrode is given by $\phi =-\mu_{Li}/e$ [34].

#### 2.2.3. Rate Constant Equation

The general rate constant equation for the events of diffusion, intercalation, and deintercalation is given by the following equation, according to reference [35]:

(12) $$\Gamma =v_{0}\;exp\;\left[-\frac{{\Delta^{*}}_{\sigma }+\alpha (H_{F}-H_{I})}{k_{B}T}\right]\;$$

In this equation, $v_{0}$ is a pre-exponential factor, α is a symmetry parameter, and ${\Delta^{*}}_{\sigma }$ is the height of the barrier for the event σ. In the present work, σ=int is the symbol for intercalation and deintercalation, and σ=diff for diffusion events. $H_{F}$ and $H_{I}$ are the Hamiltonian for the final and initial configurations, respectively; $k_{B}$ is the Boltzmann constant and T is temperature.

The Hamiltonian H, given by Equation (13a), consists of a potential energy term U and a chemical potential term μ for the sites located at the interface, i.e., for the intercalation and deintercalation events (σ=int) marked with 2 and 3 in Figure 3b.

(13a) H=U−μ

For diffusion events (σ=diff), the Hamiltonian only depends on the potential energy term:

(13b) H=U

#### 2.2.4. Potential Calculation and kMC Algorithm

Since the potential of the electrode depends on the number of ions and holes at the interface, we are interested in the rate constants of the incoming ions $\Gamma_{in}$ and the outgoing ions $\Gamma_{out}$ for the calculation of the electrode potential. Also, since for an intercalation event, $\Gamma_{in}$, $H_{I}=0$ and for a deintercalation event, $\Gamma_{out}$, $H_{F}=0$, Equation (12) yields the following:

(14) $$\Gamma_{in}=v_{0}exp\left(-\frac{{\Delta^{*}}_{int}}{k_{B}T}-\frac{\alpha (U-\mu )}{k_{B}T}\right)\;$$

and

(15) $$\Gamma_{out}=v_{0}exp\;\left(-\frac{{\Delta^{*}}_{int}}{k_{B}T}+\frac{\alpha (U-\mu )}{k_{B}T}\right)\;$$

Based on charge neutrality, for each ion being intercalated, an electron flows into the system. Conversely, each ion being deintercalated results in an electron getting out of the system. Thus, the constant electronic current applied stems from the balance between the deintercalation and the intercalation of ions. This balance leads to the following equation:

(16) $$I_{c}=e\left(\sum_{i}^{N_{fill}}\Gamma_{out}-\sum_{i}^{N_{hole}}\Gamma_{in}\right)$$

where $N_{fill}$ is the number of sites occupied by ions and $N_{hole}$ is the number of holes at the interface. Note that intercalation/deintercalation involves a negative/positive current. This is the usual convention in current electrochemistry. Replacing (14) and (15) in Equation (16) yields the following:

(17) $$I_{c}=e\left\{\sum_{i}^{N_{fill}}v_{0}exp\left(-\frac{{\Delta^{*}}_{int}}{k_{B}T}+\frac{\alpha \left(U_{i}-\mu \right)}{k_{B}T}\right)-\sum_{i}^{N_{hole}}v_{0}exp\;\left(-\frac{{\Delta^{*}}_{int}}{k_{B}T}-\frac{\alpha \left(U_{i}-\mu \right)}{k_{B}T}\right)\right\}$$

Splitting the exponential terms and taking $v_{0}$ and the exponential term containing ${\Delta^{*}}_{i}$ outside the curly brackets, we obtain the following:

(18) $$I_{c}=ev_{0}exp\;\left(-\frac{{\Delta^{*}}_{int}}{k_{B}T}\right)\;\left\{\sum_{i}^{N_{fill}}\left[exp\left(\frac{\alpha U_{i}}{k_{B}T}\right)exp\left(-\frac{\alpha \mu }{k_{B}T}\right)\right]-\sum_{i}^{N_{hole}}\left[exp\left(-\frac{\alpha U_{i}}{k_{B}T}\right)exp\left(\frac{\alpha \mu }{k_{B}T}\right)\right]\right\}$$

In a Langmuirian model, all the sites are energetically equivalent and we may set U=0. Further assuming α=0.5, Equation (18) reduces to the following:

(19) $$I_{c}=ev_{0}exp\;\left(-\frac{{\Delta^{*}}_{int}}{k_{B}T}\right)\;\left\{N_{fill}exp\left(-\frac{\mu }{2k_{B}T}\right)-N_{hole}exp\left(\frac{\mu }{{2k}_{B}T}\right)\right\}$$

An improvement to the non-interacting system should consider the interaction between the intercalated ions. This can be performed with different approaches. For example, a simple pairwise potential can be considered, up to first or second neighbors (or even beyond), or more sophisticated potentials such as the embedded-atom method or the Reactive Force Field (ReaxFF) may be used. The expected impact is a more complex behavior of the configurational entropy than in the case of the non-interacting model, which shows an entropy behavior like that of the ideal lattice-gas.

At this point, a numerical method can be used to find roots to obtain the μ value, after calculating the summation of the exponential terms for each time step. We applied the Brent’s method for this aim [36]. Then, the potential of the working electrode is obtained from ϕ=−μ/e.

**Scheme 1:**
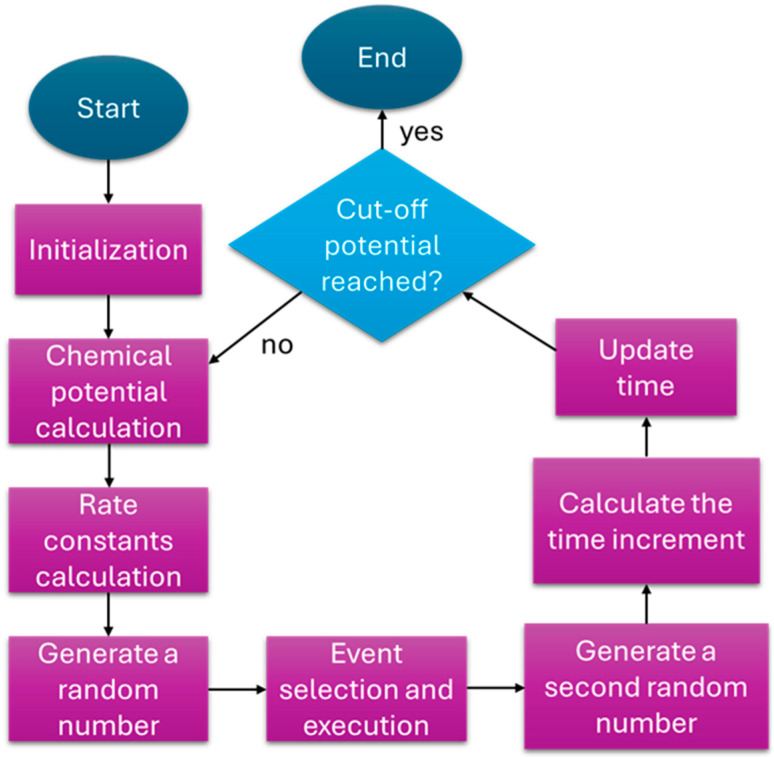
Workflow of the present kMC approach for simulating a potentiometric experiment.

The algorithm constructed for simulating galvanostatic kMC simulations consists of the following steps, detailed in the workflow of Scheme 1.

(1) Set an initial configuration and define a constant current signal (or C-rate).
(2) Estimate the chemical potential with a root-finding method using Equation (18) or Equation (19).
(3) Calculate all rate constant events with Equation (12).
(4) Generate a random number $\xi_{1}$.
(5) Choose one event using $\xi_{1}$.
(6) Perform the selected event.
(7) Generate a second random number $\xi_{2}$.
(8) Calculate the time increment with Equation (11).
(9) Update the time.
(10) Repeat steps from 2 to 9 until reaching the cut-off electrode potential.

The output quantities for each simulation are averaged for the mean average time between two time points $t_{j}=(t_{i}+t_{i+1})/2$ provided that $t_{i}<t\leq t_{i+1}$.

### 2.3. Calculating Continuum Parameters from Microscopic Quantities

#### 2.3.1. Diffusion Coefficient

In a 3D lattice, the diffusion coefficient may be calculated from [37]:

(20) $$D=\frac{1}{6}\Gamma_{tot}a^{2}$$

where a is a characteristic diffusion length and $\Gamma_{tot}$ is the sum of rate constants calculated with Equation (12) for all independent diffusion events ($\Gamma_{i}$ in Equation (21b)) of single ions in the lattice.

(21a) $$\Gamma_{tot}=\sum_{i}^{n}\Gamma_{i}$$

(21b) $$\Gamma_{i}=v_{0}exp\;\left[-\frac{{\Delta^{*}}_{diff}+\alpha (U_{F,i}-U_{I})}{k_{B}T}\right]$$

If no interactions are considered, Equation (21b) yields the following:

(21c) $$\Gamma_{i}=v_{0}exp\left(-\frac{{\Delta^{*}}_{diff}}{k_{B}T}\right)$$

That is the rate constant for diffusion events used in the present work.

In a simple cubic lattice, the n = 6 nearest neighbors method presents the same rate for diffusion, and so Equation (20) yields the following:

(22) $$D=v_{0}a^{2}exp\left(-\frac{{\Delta^{*}}_{diff}}{k_{B}T}\right)$$

This is the same expression derived by [22,24,38] under Langmuir conditions.

For isotropic systems, such as cubic crystals, the displacements in x, y, and z are the same, and therefore we obtain the following:

(23) $$\langle x^{2}\rangle =\langle y^{2}\rangle =\langle z^{2}\rangle =\frac{1}{3}\langle R^{2}\rangle$$

Here, $\langle R^{2}\rangle$ is the mean square displacement. Then, the diffusion coefficient in the y-direction is as follows:

(24) $$D_{y}=D=v_{0}a^{2}exp\left(-\frac{{\Delta^{*}}_{diff}}{k_{B}T}\right)$$

The continuum diffusion length d was calculated with the number of lattice sites in this direction and with the jump distance, i.e., $d=L_{y}=N_{y}a$. The surface in contact with the electrolyte was calculated as $S=N_{x}N_{z}a^{2}.$

#### 2.3.2. Charge Transfer Rate Constant

According to the literature [39], the rate constants of the forward and reverse processes of a redox reaction like that given in Equation (1) are derived from the following equations:

(25) $$k_{f}=A_{f}exp\left(-\frac{{\Delta G}_{c}^{\neq }}{RT}\right)$$

(26) $$k_{b}=A_{b}exp\left(-\frac{{\Delta G}_{a}^{\neq }}{RT}\right)$$

**Figure 4:**
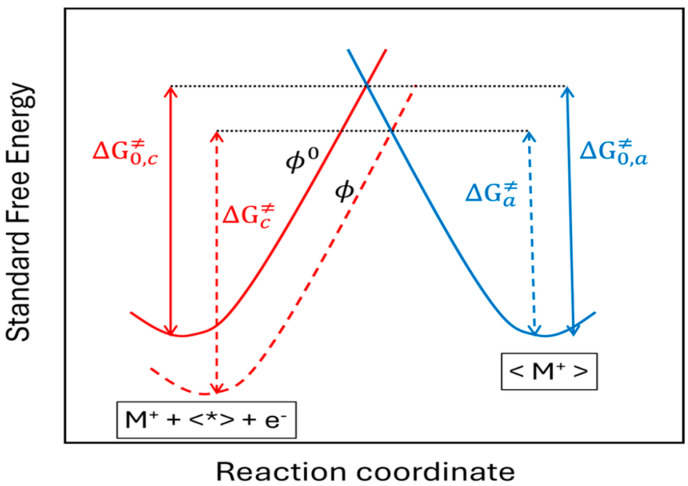
Reaction coordinate for the intercalation phenomena when the potential of the working electrode is equal to the standard equilibrium potential $\phi^{0}$ and when the working electrode potential is different from that of the equilibrium ϕ. The reaction in the figure corresponds to Equation (1).

Here, ${\Delta G}_{c}^{\neq }$ and ${\Delta G}_{a}^{\neq }$ are the free energy barriers for reduction and oxidation, respectively (Figure 4). The changes in these barriers with the displacements from the equilibrium potential, $\phi^{0}$, can be expressed as follows:

(27) $${\Delta G}_{c}^{\neq }={\Delta G}_{0,c}^{\neq }-(1-\alpha )(\phi -\phi^{0})$$

(28) $${\Delta G}_{a}^{\neq }={\Delta G}_{0,a}^{\neq }+\alpha (\phi -\phi^{0})$$

Here, ${\Delta G}_{0,c}^{\neq }$ y ${\Delta G}_{0,a}^{\neq }$ corresponds to the free energy barriers when the electrode potential $\phi =\phi^{0}$ (Figure 4). Replacing the values in Equations (25) and (26) yields the following:

(29) $$k_{f}=A_{f}exp\left(-\frac{{\Delta G}_{0,c}^{\neq }}{RT}\right)exp\left(\frac{-\alpha F(\phi -\phi^{0})}{RT}\right)$$

(30) $$k_{b}=A_{b}exp\left(-\frac{{\Delta G}_{0,a}^{\neq }}{RT}\right)exp\left(\frac{\left(1-\alpha )F(\phi -\phi^{0}\right)}{RT}\right)$$

From these last equations, it emerges that when equilibrium is reached ($\phi =\phi^{0}$), $k_{f}=k_{b}=k^{0}$, and so Equations (29) and (30) yield the following:

(31) $$k_{f}=k^{0}exp\left(\frac{-\alpha F(\phi -\phi^{0})}{RT}\right)$$

(32) $$k_{b}=k^{0}exp\left(\frac{\left(1-\alpha )F(\phi -\phi^{0}\right)}{RT}\right)$$

From these last equations arises the following:

(33) $$k^{0}=A_{f}exp\left(-\frac{{\Delta G}_{0,c}^{\neq }}{RT}\right)=A_{b}exp\left(-\frac{{\Delta G}_{0,a}^{\neq }}{RT}\right)$$

This gives the Butler–Volmer equation at the macroscopic level [40]:

(34) $$I=SFc_{max}k^{0}\left[xexp\left(\frac{\left(1-\alpha )F(\phi -\phi^{0}\right)}{RT}\right)-(1-x)exp\left(-\frac{\alpha F(\phi -\phi^{0})}{RT}\right)\right]$$

An equivalence can be established with the microscopic description given in Section 2.2.3. From the comparison between Equations (18) and (34), it emerges that the following term

(35) $$\Gamma^{0}=v_{0}exp\left(-\frac{{\Delta^{*}}_{int}}{k_{B}T}\right)$$

is equivalent to the rate constant $k^{0}$, but in s^−1^ units.

In a previous work [27], we derived an expression to estimate the rate constant of the Butler–Volmer equation from the microscopic rate constants. It is just a matter of multiplying the microscopic rate constant by a volume/area parameter, i.e., as follows:

(36) $$k^{0}=\Gamma^{0}d^{0}={d^{0}v}_{0}exp\left(-\frac{{\Delta^{*}}_{int}}{k_{B}T}\right)$$

where

(37) $$d^{0}=\frac{volume}{surface}$$

The parameter $d^{0}\;is\;calculated\;as$ the unit cell volume divided by the unit cell surface that contains one single site of the lattice.

## 3. Results and Discussion

The code for kMC simulations was written in C++ and was compiled with gcc 12.3.0 and was parallelized with OpenMP. Continuum SPM simulations were performed using the open-source software *galpynostatic* v 0.5.13, with the module *simulation*, available at https://github.com/fernandezfran/galpynostatic, accessed on 29 April 2025 [30]. In all cases, Langmuir intercalation isotherms were considered. In the kMC simulations, the diffusion jump distance of the simple cubic lattice was kept constant at a=1Å, and then $d^{0}$ = 1Å and the pre-exponential factor was as assumed as $v_{0}=1\;\times \;10^{13}\;s^{-1}$; this is a value commonly used for intercalation materials, which was calculated for graphite [41]. It is important to note that this is taken as a typical value. However, in the context of specific materials, $v_{0}$ may be different.

The first validation test of the galvanostatic kMC code was to check the expected linear relationship between the SoC and the simulated time (see Equations (7) and (8b)), which yields the following:

(38) $$SoC=\frac{I_{c}}{Qmax}\;t$$

**Figure 5:**
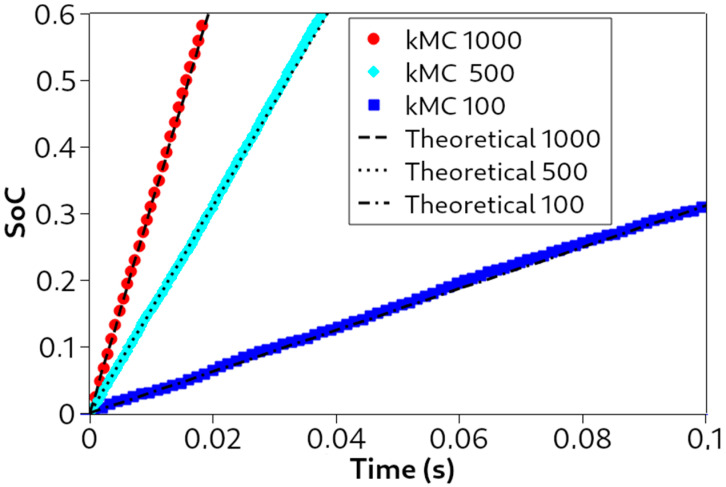
SoC increment with time for three different current densities: −100 mA/cm^2^, −500 mA/cm^2^, and −1000 mA/cm^2^. The figure shows the kMC results (with symbols) and the theoretical calculations (lines).

This is typical for galvanostatic experiments. As observed in Figure 5, the kMC simulations (represented with symbols) follow the expected theoretical trends (represented with lines), where an increase in current density, from −100 mA/cm^2^ to −1000 mA/cm^2^, increases the slope of the linear behavior. In these simulations, only the current density was varied, keeping the rest of the parameters constant.

Next, the agreement between the kMC and continuum potential/SoC profiles obtained after applying a constant current signal was tested. With this purpose, kMC barriers were directly estimated from the macroscopic parameters at the continuum scale. This was calculated from the relationships provided in Section 2.3. For example, let us consider two intercalation materials A and B defined by the set of continuum parameters (D, $k^{0}$, d, and $C_{r}$) given in Table 1, with cubic simulation boxes of $N_{T}=20\times 20\times 20$ for system A and 30×30×30 for B. To calculate the energy barriers for diffusion and intercalation, Equations (24) and (36) were used, respectively. The values obtained for the barriers ${\Delta^{*}}_{int}$ and ${\Delta^{*}}_{diff}$ are provided in Table 1.

**Figure 6:**
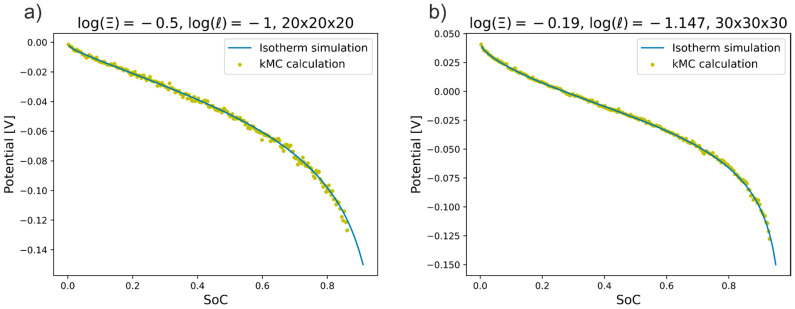
Comparison of two selected cases of the galvanostatic transients obtained from the continuum simulations with the method described in Section 2.1 (lines) and the kMC calculations (dots). The parameters are given in Table 1. The log(Ξ), log(l) values and cell size implemented are given above figures (**a**,**b**).

Figure 6 shows the potential/SoC profiles for materials A and B obtained from the numerical simulations (blue line) and kMC calculations (yellow dots) for one kMC run. The kMC simulations show fluctuating behavior around the continuum value. However, despite the restricted statistics of a single kMC run, the two methods show excellent agreement.

To check that the parameters selected correspond to relevant cases of the problem under consideration, the scalable parameters Ξ and l for these materials were estimated with Equations (9) and (10). The calculated dimensionless values are detailed in Table 1 and at the top of Figure 6. This allows the materials to be located on the galvanostatic map, introduced in Figure 2b. In the two cases presented, the materials reach a similar SoC when the cut-off potential is reached; however, material A allows for a charging rate three times greater than material B, which would be beneficial for having faster-charging batteries.

From now on, we will report the values of the scalable parameters Ξ and l instead of detailing the whole dataset, as well as the information necessary for the analysis.

### 3.1. Number of Samples and Simulation Box Size 

In this section, we will analyze how the number of independent samples (number of kMC runs) and the size of the simulation box impact the potential profiles.

**Figure 7:**
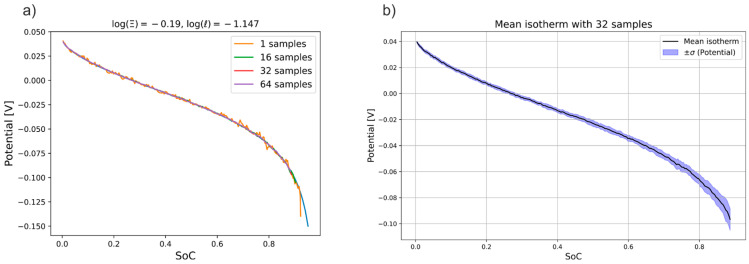
Statistical analysis of the kMC calculations vs. the theoretical isotherm at log(Ξ)=−0.19 and log(l)=−1.147 and 20 × 20 × 20 box size. (**a**) The average kMC curve as a function of the sample number; (**b**) potential standard deviation as a function of SoC for 32 samples.

**Figure 8:**
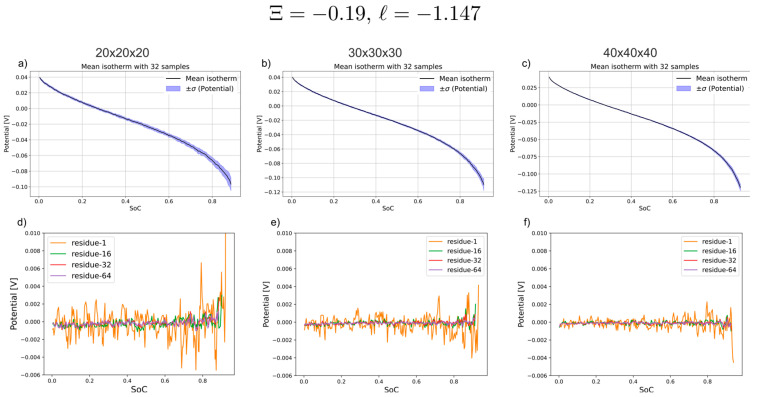
(**a**–**c**) Convergence analysis of the kMC calculations as a function of the box size. As the surface area increases, the mean kMC curve converges to the theoretical curve and the standard deviation diminishes. (**d**–**f**) Residual analysis of the convergence of the kMC results to the theoretical isotherm. As the number of sites increases, the residuals become smaller.

Figure 7 shows the comparison between the potential/SoC profiles obtained from the kMC simulations with different numbers of statistical samples and the continuum simulations for the same Ξ and l point (log(Ξ)=−0.19 and log(l)=−1.147). As shown before, a single kMC sample already shows a random dispersion close to the Langmuir prediction, Figure 7a. As the number of samples increases, the disperse behavior becomes smoother and the results closely coincide. This is better shown in Figure 8d when plotting the residuals for different numbers of samples. As observed, the results with 32 and 64 samples are practically the same. Figure 7b shows the average curve (black line) with 32 samples. The standard deviation of the potential was calculated (blue shadow area). The deviation increases for larger SoC values. This latter fact is understandable, since as the SoC increases, the number of available sites for particle insertion becomes smaller, which makes the outcome for the potential more dispersed.

Figure 8 shows an analysis of the dispersion for the predicted potential as a function of box size using 32 samples, for the same Ξ and l point as that of the previous case. It can be seen that increasing the number of sites leads to a decrease in the fluctuations of the potential, making the standard deviation much lower. Therefore, increasing the size of the box helps to reduce the number of samples required to obtain a converged isotherm. This effect becomes more remarkable at larger SoCs, where the number of free surface sites where the ions may be intercalated sinks.

A residual analysis was performed for these box sizes varying the number of samples. The residual between the continuum potential/SoC curves and the kMC curves is shown in Figure 8d–f. It is clear that with increasing box size, the residual values decrease for all sample numbers, leading to the improved convergence of the voltage profiles. To help understand the sample and lattice size effects, an error estimation using Equation (39) was made. The error of the kMC predictions relative to the continuum simulations was estimated from the differences:

(39) $$error=\frac{\sum_{i}\left|\phi_{i}^{kMC}-\phi_{i}^{cont}\right|}{\sum_{i}\left|\phi_{i}^{cont}\right|}\;✕\;100\;$$

where the upper indices kMC and cont correspond to the results of the kMC and continuum simulations, and the index i runs over the simulated kMC points. Table 2 shows the errors as a function of the number of samples and box size.

### 3.2. Limitation by the Number of Surface Sites

**Figure 9:**
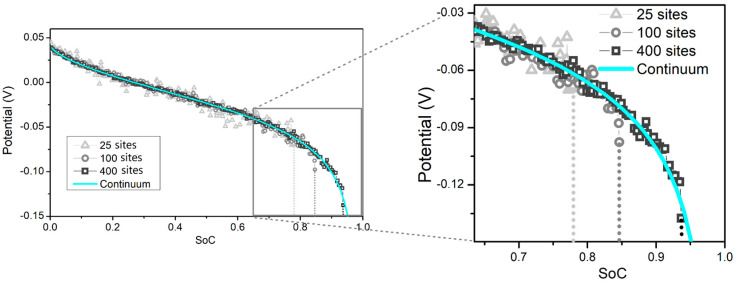
Numerical and kMC voltage profiles for three different areas at the electrode/electrolyte interface, 5 × 5 (25 sites), 10 × 10 (100 sites), and 20 × 20 (400 sites), for the same number of sites at the *y*-axis, 20 sites. The point considered was log(Ξ)=−0.19 and log(l)=−1.147 in all cases. Dashed lines were added with the same color of the simulation box considered to represent the sudden decrease in potential that crosses the potential cut-off of −0.15 V.

In most of the cases displayed above, it was observed that the kMC potential profiles present a drop that reaches the potential cut-off (−0.15 V) at a SoC value that is lower than the final SoC of the numerical simulations. Although not obvious in the representations shown before, this drop is evident in Figure 9, for three different discretizations of the surface at the same (Ξ,l) point, log(Ξ)=−0.19 and log(l)=−1.147. At this (Ξ,l) point the rate-controlling process is charge transfer, according to the details given in Figure 2b. As observed, something is preventing the SoC of kMC from reaching its numerical counterpart.

Figure 9 shows results for three different discretizations of the simulation box, keeping constant the number of sites $N_{y}=20$, and varying the number of surface sites $N_{x}\;$ × $N_{z}$ = 25 (5 × 5), 100 (10 × 10) and 400 (20 × 20), for the same Ξ and l point. As observed, for a single kMC sample, an increase in the number of sites in contact with the electrolyte causes a shift in the potential profile towards higher SoCs, approaching the results of the continuum simulations. This is understood from the previous comments: if the number of lattice sites at the interface is small, the fluctuations in the electrode potential will be larger with an increasing number of occupied surface sites. This results in a potential jump that exceeds the cut-off potential at a SoC value lower than the continuum value.

It is important to remark that changing the number of sites $N_{T}$, and so $Q_{max}$, leads to different continuum parameters for the same Ξ and l point, according to the equations seen in Section 2.1. These parameters are given in Table 3.

**Table 3:** Parameters corresponding to data shown in Figure 9 and Figure 10.

Box Size ($N_{x}\times N_{y}\times N_{z}$)	d [Å]	$C_{r}$	$Q_{max}$ [C]	$D_{y}$ [cm^2^/s]	$k^{0}$ [cm/s]	$\Delta_{diff}^{*}$ [eV]	$\Delta_{int}^{*}$ [eV]	[log(Ξ), log(*l*)]
5 × 20 × 5	20	22,454	8.01 × 10^−17^	3.5 × 10^−12^	3.02 × 10^−6^	0.5	0.62	[−0.19, −1.147]
10 × 20 × 10	20	22,454	3.2 × 10^−16^	3.5 × 10^−12^	3.02 × 10^−6^	0.5	0.62	[−0.19, −1.147]
20 × 20 × 20	20	22,454	1.15 × 10^−15^	3.5 × 10^−12^	3.02 × 10^−6^	0.5	0.62	[−0.19, −1.147]
60 × 20 × 60	20	203,381	1.15 × 10^−14^	3.5 × 10^−12^	2.23 × 10^−5^	0.5	0.57	[−0.19, 0.2]
60 × 40 × 60	40	50,845	2.31 × 10^−14^	3.5 × 10^−12^	1.14 × 10^−5^	0.5	0.59	[−0.19, 0.2]
60 × 60 × 60	60	22,597	3.46 × 10^−14^	3.5 × 10^−12^	7.42 × 10^−6^	0.5	0.60	[−0.19, 0.2]

In summary, there is a discretization effect due to the finite number of sites at the interface where ion exchange occurs. Therefore, the interface between the electrolyte and the electrode must contain enough sites to approach the continuum behavior when modeling galvanostatic profiles with kMC.

### 3.3. Limitation by Finite-Size Diffusion

Another source of discrepancy between the kMC potential profiles and the continuum curves appears when mass transport becomes rate-controlling. An analysis of the convergence of the kMC and the theoretical isotherms by varying the number of sites in the *y* direction, say $N_{y}$, was carried out. For this, a (Ξ,l) point corresponding to [log($\Xi ),log\left(l\right)]=[$−0.19, 0.2] was used, corresponding to the case where the SoC drop is associated with diffusional limitations (see the lines in Figure 2 and point c in Figure 11 and the discussion in reference [4]).

**Figure 10:**
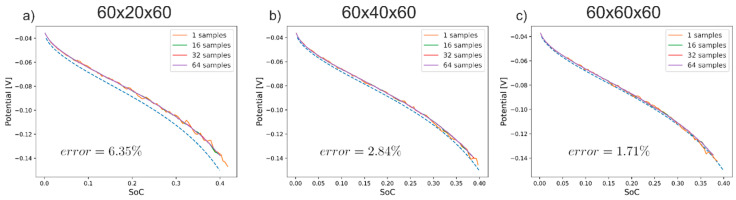
Impact of the box size on the kMC convergence in the diffusion control zone. As the box depth increases ($N_{y}$), the error between the curves diminishes for the same area $N_{x}\times N_{z}$. (**a**) $N_{y}$ = 20 (**b**) $N_{y}$ = 40 (**c**) $N_{y}$ = 60.

Figure 10 compares the kMC and continuum results. As the number of points used to discretize in the *y* direction increases from $N_{y}$ = 20 (Figure 10a) to $N_{y}$ = 40 (Figure 10b) and $N_{y}$ = 60 (Figure 10c), for the same surface area sites ($N_{x}\;$× $N_{z}$ = 60 × 60), the relative difference between the curves decreases. The errors between the kMC and continuum curves were calculated with Equation (39) with the maximum number of kMC samples (64) and are indicated in each of the panels of the figure.

We remark again that changing the number of sites of the simulation box would lead to different continuum parameters for the same Ξ and l point, according to the equations seen in Section 2.1. This is shown in Table 3.

From the present analysis, it can be deduced that to obtain the correct voltage profiles, provided that there is some degree of diffusional control, the indicated number of sites in the direction of diffusion must be considered.

### 3.4. Validation of kMC with the Galvanostatic Map

As discussed in the section “Scalable Parameters at Galvanostatic Conditions,” different regions in the galvanostatic map are related to different types of control over the kinetics of intercalation. Therefore, this map serves as a guide to check that the kMC simulations reproduce the behavior of continuum models under different types of kinetic limitations. Thus, an analysis of the kMC behavior at different [log(Ξ), log(l)] couples was made.

**Figure 11:**
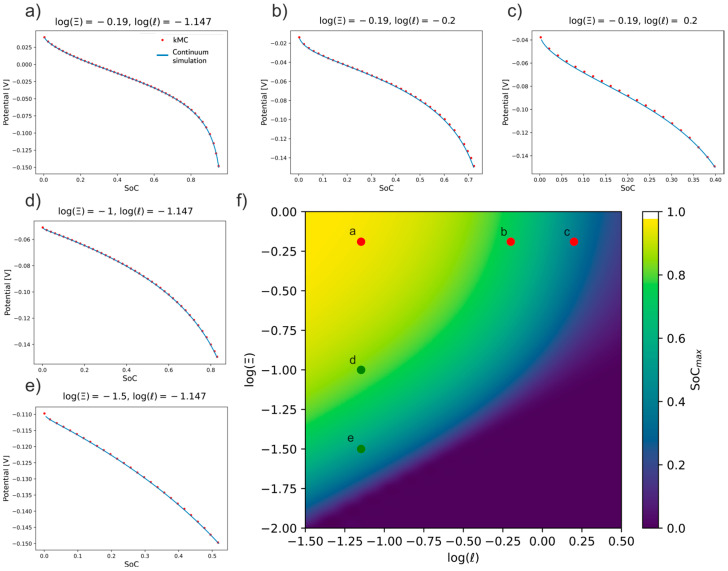
Different kMC curves simulated with 64 samples compared to the continuum simulations and their respective positions on the SoC map. The voltage profiles shown in figures (**a**–**e**) corresponds to the simulations of the points placed in the map of figure (**f**).

Figure 11f shows different points on the map selected to test the kMC simulations, with the corresponding galvanostatic transient given in Figure 11a–e. The kMC models for each of the points were constructed taking into account the limitations previously evaluated in Section 3.1 and Section 3.2. All kMC voltage/SoC profiles show an excellent agreement with the continuum calculations.

The reference point to analyze the simulations was point a in Figure 11f, (log(Ξ)=−0.19, log(l)=−1.147). At this point, the maximum SoC is practically reached since there is no kinetic limitation, due to a large $k^{0}/{C_{r}}^{1/2}$ ratio, and diffusion kinetics is relatively fast, due to a small $d^{2}/D$ ratio. The kMC-point-a.mp4 video in the Supplementary Information (SI)  shows the evolution of this simulation over time. In all the videos, the interphase electrode/electrolyte is located on the left-hand side and the diffusion limit is on the right-hand side of the screen. Furthermore, the ions located at the interface are represented with a different color.

When the ordinate is fixed at log(Ξ)=−0.19 and log(l) is increased from the reference point, the system enters into the diffusion control zone of the map, the transients of Figure 11b,c. The video kMC-point-c.mp4 in the SI shows the evolution of the system at point c over time. It is remarkable how the intercalated ion front does not reach the inner part of the electrode.

On the other hand, when the abscissa is fixed at log(l)=−1.147 and log(Ξ) is decreased from point a, the map approaches the zone regulated by charge transfer, with the transients given in Figure 11d,e. In the file kMC-point-e.mp4 in SI, the evolution of the frames at point e over time shows that although the ions cover the entire length of the slab, the slab is not fully occupied with Li-ions. This is so because there is a slow exchange of ions at the interface.

This is a key result for our work since it demonstrates that the kMC model works correctly under different kinetic regimes of the lithiation process, which consolidates the robustness of the kMC model. It also demonstrates the validity of the equations that connect the parameters of the continuum with those of the microscopic level, on the basis of the galvanostatic map developed by us previously [4,5] as a guide to easily deal with a problem with numerous variables to handle.

Finally, we added in the Supplementary Information a section where we discuss the scope of the assumption made in Section 2.2.2, that the concentration of ions in the electrolyte remains constant. There, we show that the C-rates applied in the present work are too slow to cause considerable variations in the electrolyte concentration. A benchmark of the code was also performed by analyzing the calculation time depending on the number of sites and the number of threads. The results of this analysis are shown in the Supplementary Information.

## 4. Conclusions and Perspectives

We have shown that kinetic Monte Carlo simulations can replicate the physics of macro-scale intercalation systems under galvanostatic conditions. This bridges the gap between the electrochemistry of battery materials at macro and micro levels, allowing us to perform computational experiments of potential profiles with microscopic details of the events.

An intercalation Langmuir isotherm was used for both numerical and kMC simulations, as well as the kMC algorithm considering a galvanostatic signal. Also, we have shown how the kMC parameters are linked to those of the continuum equations. In particular, we have shown how the microscopic parameters can be controlled from those of the continuum level.

We have shown the importance of performing tests to avoid problems due to the discretization of the systems, as well as to evaluate the number of statistical samples necessary to obtain converged potential/State-of-Charge profiles. The validity of the kMC simulations was tested using a theoretical framework developed previously by us, consisting of maps that use two scalable parameters that determine the galvanostatic profiles. The good agreement between both approaches demonstrated the validity of the Monte Carlo modeling.

This work opens a door to explore the kinetics of alkali-metal ion intercalation materials using more realistic modeling to represent the interaction among inserted ions. In particular, this approach may allow us to address problems that arise due to the limitations of continuum models to cover some physical aspects of intercalation materials, for example, phase coexistence, entropic factors arising from the configurations of the intercalated ions, the solid-state mass transport inside the electrodes, and the geometry of the solid material, just to name a few factors.

## Figures and Tables

**Table 1:** Values of log(Ξ), log(l), *d*, and $D_{y}$ selected to perform the comparison between the continuum and kMC simulations. The corresponding $k^{0},$ C-rate, ${\Delta^{*}}_{diff}$, and ${\Delta^{*}}_{int}$ are also reported.

Material	$Q_{max}$ [C]	d [Å]	$C_{r}$	$D_{y}$ [cm^2^/s]	$k^{0}$ [cm/s]	${\Delta^{*}}_{diff}\;\left[eV\right]$	${\Delta^{*}}_{int}\;\left[eV\right]$	log(Ξ)	log(l)
A	1.282 × 10^−15^	20	31,500	3.5 × 10^−12^	$1.75\times 10^{-6}$	0.5	0.636	−0.5	−1
B	4.325 × 10^−15^	30	9980	3.5 × 10^−12^	$2.01\times 10^{-6}$	0.5	0.640	−0.19	−1.147

**Table 2:** Errors calculated with Equation (39), in %, as a function of the box size and sample number.

Samples/Size	20 × 20 × 20	30 × 30 × 30	40 × 40 × 40
1	4.11	2.22	1.45
16	1.35	0.68	0.51
32	1.05	0.52	0.41
64	0.96	0.42	0.37

## Data Availability

Dataset available on request from the authors.

## Supplementary Materials

The following supporting information can be downloaded at: https://www.mdpi.com/article/10.3390/e27070663/s1.

## Abbreviations

The following abbreviations are used in this manuscript:

kMC	Kinetic Monte Carlo
LIBs	Lithium-ion batteries
SPM	Single-particle model
SoC	State-of-Charge
SI	Supplementary Information
