# Supplementary material for: Electrochemical Modeling Applied to Intercalation Phenomena Using Lattice Kinetic Monte Carlo Simulations: Galvanostatic Simulations
Source: Entropy (Basel). 2025 Jun 20;27(7):663. doi: 10.3390/e27070663 (PMC12293983; doi:10.3390/e27070663)
Supplement: Supplementary file 1 [file entropy-27-00663-s001.zip › entropy-3648340-supplementary.pdf]

Supplementary Information:

Electrochemical Modelling Applied to Intercalation Phenomena Using Lattice Kinetic Monte Carlo Simulations: Galvanostatic Simulations

E. Maximiliano Gavilán-Arriazu[1,2*], Andrés Ruderman[2,3], Carlos Bederian[3], Eduardo Moran Vieyra[1], Ezequiel P. M. Leiva[2,4*]

[1] Instituto de Bionanotecnología del NOA (INBIONATEC), Universidad Nacional de Santiago del Estero (UNSE), G4206XCP Santiago del Estero, Argentina
[2] Laboratorio de Energías Sustentables, Facultad de Matemática, Astronomía, Física y Computación, Universidad Nacional de Córdoba, Córdoba, Argentina
[3] Instituto de Física Enrique Gaviola, Consejo Nacional de Investigaciones Científicas y Técnicas (CONICET), Córdoba, Argentina
[4] Instituto de Fisicoquímica de Córdoba (INFIQC), Consejo Nacional de Investigaciones Científicas y Técnicas (CONICET), Córdoba, Argentina

*Correspondence: E.P.M.L: ezequiel.leiva@unc.edu.ar and E.M.G: maxigavilan@gmail.com

S1 – Benchmarking kMC simulations with a galvanostatic algorithm

All calculations were performed on the *Serafin* cluster of the Centro de Computación de alto Desempeño (https://supercomputo.unc.edu.ar/) from the Universidad Nacional de Córdoba (UNC). *Serafin* comprises 60 compute nodes, each with the following characteristics: Two AMD EPYC 7532 processors with 32 cores each (64 cores in total per node) with Zen 2 architecture; 128 GB of DDR4-3200 RAM, arranged in 16 modules of 8 GB; 1.92 TB of Samsung PM983 M.2 NVMe SSD storage. It also features Infiniband HDR100 and an NVIDIA MCX653105A-ECAT-SP ConnectX-6 network board.

These specifications add up to a total of 3840 computing cores and a peak performance of 147 TFLOPS at double precision (float64).

Additionally, there is an interactive compute node equipped with a 32-core AMD Ryzen Threadripper PRO 3975WX processor, 128 GB of RAM, and an Infiniband FDR board for data analysis and visualization.

Here we report the simulation times for different cubic box sizes and number of threads. **Figure S1** shows the results for a simulation box of 30x30x30 varying the number of parallelization threads. We set the maximum number of threads to 16 since we require several statistical samples. This means that we have sacrificed improving the computing time of an individual simulation in order to run many simulations in parallel and make better use of computational resources. As observed, the computational time decreases when increasing the number of threads. **Figure S2** displays the increase in computational time when varying the size of the cubic box using 4 threads.

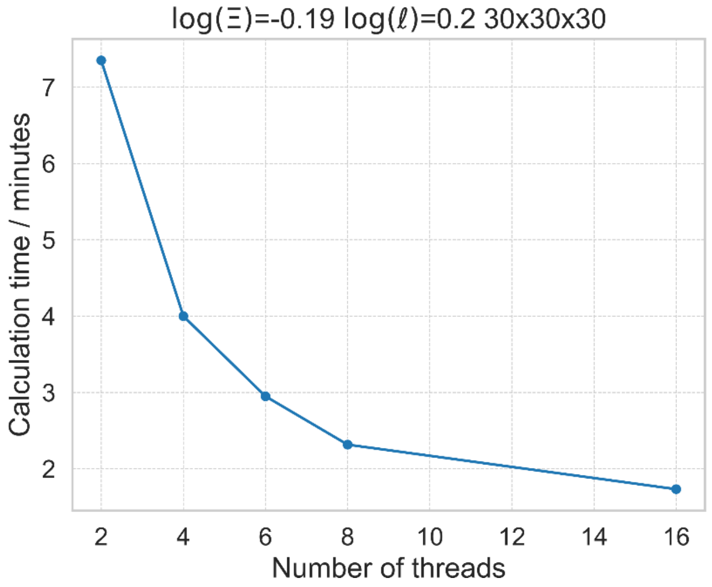

Figure S1. Simulation time for different kMC simulations with different number of threads.

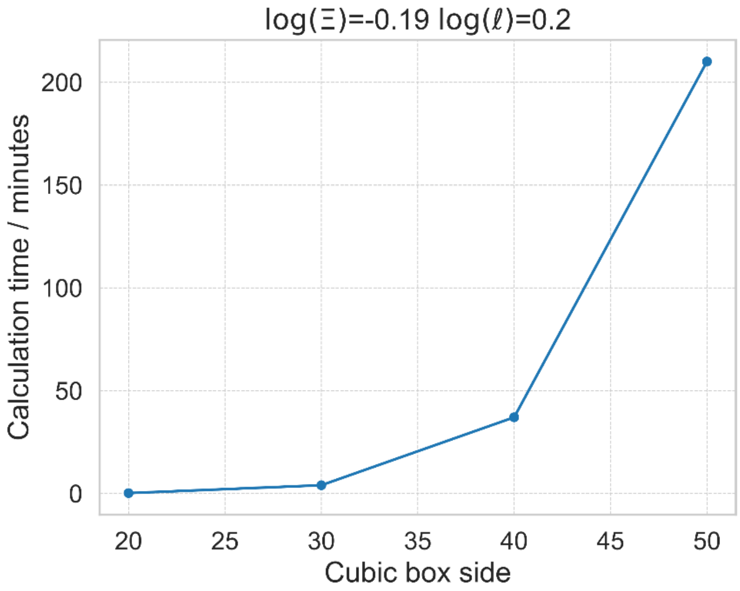

Figure S2. Simulation time for different kMC simulations with different cubic box sizes.

It is important to remark that the computational time depends on the considered $\Xi - l$ point. It is to be expected that for larger final SoCs (for a given potential cut-off) the computational time increases.

S2 - Limitations of the constant electrolyte concentration assumption

Analyzing the limitation of assuming a constant concentration of Li^+ in the electrolyte can be done by estimating the maximum C-rate that may be applied to the electrochemical cell before an important depletion of electrolyte concentration at the electrode/electrolyte interface. With this purpose, we consider that diffusion is governing electrolyte mass transport.

Let us assume that the ions concentration at the electrolyte solution (c_e) follows a 1D linear diffusion, given by second Fick's law:

$$\frac{\partial c_e}{\partial t} = D_e \frac{\partial^2 c_e}{\partial x^2} \tag{S1}$$

Where D_e is the diffusion coefficient of Li^+ ions in the electrolyte.
With the boundary condition:

$$\left(-D_e \frac{\partial c_e}{\partial x}\right)_{x=0} = \frac{j}{F} \tag{S2}$$

at the electrolyte / electrode interface, where j is the current representing the sink of ions at the surface of the electrode. and

$$c_e(x \to \infty. t) = c_{e,0} \tag{S3}$$

at the bulk of the solution.
Also, initially

$$c_e(x.0) = c_{e,0} \qquad \text{(S4)}$$

Since the current is constant over all the experiment (galvanostatic conditions), this is a classical problem of a constant flux at the boundary x=0 (electrode / electrolyte interface). The analytical solution for the concentration at the interface is [1]:

$$c_e(0.t) = c_{e,0} - \frac{2j}{F}\left(\frac{t}{\pi D_e}\right)^{1/2} \qquad \text{(S5)}$$

Now, let us consider a small concentration drop at the interface, say 5% below its initial value, so that the income of ions into the electrode material is only slightly perturbed. This involves the condition:

$$c_e(0.t) = (1-\sigma)\, c_{e,0} \qquad \text{(S6)}$$

with $\sigma = 0.05$
Thus, this depletion will occur at the time t_σ

$$(1-\sigma)c_{e,0} = c_{e,0} - \frac{2j}{F}\left(\frac{t_\sigma}{\pi D_e}\right)^{1/2} \qquad \text{(S7)}$$

$$\frac{2j}{F}\left(\frac{t_\sigma}{\pi D_e}\right)^{1/2} = \sigma c_{e,0} \qquad \text{(S8)}$$

$$t_\sigma = \left(\frac{F\sigma c_{e,0}}{2j}\right)^2 \pi D_e \qquad \text{(S9)}$$

Thus, t_σ is the time at which the electrolyte concentration at the electrolyte/electrode boundary is decreased in 5% with respect to its bulk value.
Let us now relate this time with the charging time of the electrode material, t_{charge}:

$$t_{charge} = \frac{Q_{area}}{j} \qquad \text{(S10)}$$

Which is the time at which the electrode material would be completely charged at the current density j. Where Q_{area} is the total charge per unit area. Thus, equating equations (S9) and (S10)

$$\left(\frac{F\sigma c_{e,0}}{2j}\right)^2 \pi D_e = \frac{Q_{area}}{j} \qquad \text{(S11)}$$

The meaning of this equation is the following: it gives us the current density j at which the electrolyte concentration at the interface will drop 5% relative to its bulk value when the electrode material has been completely loaded with Li^+ ions. Before that moment, the electrolyte concentration will be obviously larger.

Let us denote this current density with j_{max}, since current densities larger than this will drop the interface Li^+ ion concentration in more than 5%. From equation (S11) we get:

$$j_{max} = \frac{\left(F\sigma c_{e,0}\right)^2}{4Q_{area}}\pi D_e \qquad \text{(S12)}$$

The C-rate C_r is related to the current density by the equiation:

$$C_r = \frac{j t_h}{Q_{area}} \qquad \text{(S13)}$$

So, we can define a maximum C-rate, $C_{r,max}$ as:

$$C_{r,max} = \frac{j_{max} t_h}{Q_{area}} \qquad \text{(S14)}$$

which corresponds to the maximum C-rate that may be applied without producing a Li^+ ion interfacial concentration drops larger than 5%

Replacing in into Equation (S12)

$$C_{r,max} = \frac{\left(F\sigma c_{e,0}\right)^2}{4{Q_{area}}^2}\pi D_e t_h \qquad \text{(S15)}$$

Since

$$Q_{area} = \frac{V}{A} c_{max,s} F = d c_{max,s} F \qquad \text{(S16)}$$

Where $c_{max,s}$ is the maximum Li^+ concentration allowed in the solid material and d is the characteristic diffusion length in the material. Replacing

$$C_{r,max} = \frac{F^2\sigma^2 {c_{e,0}}^2 \pi D_e t_h}{4d^2 {c_{max,s}}^2 F^2} \qquad \text{(S17)}$$

And so

$$C_{r,max} = \left(\frac{\sigma c_{e,0}}{d c_{max,s}}\right)^2 \frac{\pi D_e t_h}{4} \qquad \text{(S18)}$$

For example, taking typical values $D_e = 1x10^{-6} cm^2/s$, $c_{e,0}/c_{max,s}$= 1 / 22 and $\sigma = 0.05$ and $d = 20\,\text{Å}, 40\,\text{Å}$ and $60\,\text{Å}$, $C_{r,max} = 365113, 91278$ and 40568 , respectively. This means that $C_{r,max}$ is always larger than those C-rates applied in our work. So, in principle, diffusional limitations in the electrolyte should be irrelevant under the present modeling conditions.
